# Respiratory bronchiolitis-interstitial lung disease

**DOI:** 10.1186/s13023-014-0106-8

**Published:** 2014-07-11

**Authors:** Alicja Sieminska, Krzysztof Kuziemski

**Affiliations:** 1 Department of Allergology and Pneumonology, Medical University of Gdansk, Debinki Str 7, Gdansk 80-211, Poland

**Keywords:** Respiratory bronchiolitis-interstitial lung disease, Smoking-related disease, Idiopathic interstitial pneumonia

## Abstract

Respiratory bronchiolitis-associated interstitial lung disease (RB-ILD) is a rare, mild inflammatory pulmonary disorder that occurs almost exclusively in current or former heavy smokers, usually between the third and sixth decades, most likely with no gender predilection. The onset is usually insidious with exertional dyspnea and persistent cough, which may be non-productive, developing over a course of weeks or months. RB-ILD may also be diagnosed in asymptomatic patients with functional impairment and chest radiograph or high-resolution computed tomography (HRCT) abnormalities. Histologically, RB-ILD is characterized by the accumulation of yellow-brown pigmented macrophages within the lumens of respiratory bronchioles and alveolar ducts, associated with a patchy submucosal and peribronchiolar chronic inflammation. Common findings also include mild bronchiolar and peribronchiolar alveolar fibrosis that expands contiguous alveolar septa and leads to architectural distortion as well as centrilobular emphysema. Chest radiographs in patients with RB-ILD typically show fine reticulonodular interstitial opacities, while on HRCT central and peripheral bronchial wall thickening, centrilobular nodules, and ground-glass opacities associated with upper lobe centrilobular emphysema are most frequently reported. Pulmonary function testing may be normal but usually demonstrates mixed, predominantly obstructive abnormalities, often combined with hyperinflation and usually associated with a mild to moderate reduction in carbon monoxide diffusion capacity (DLco). The course of RB-ILD is heterogeneous. Some patients respond favorably to corticosteroids and/or smoking cessation, but often there is no functional improvement and the disease progresses despite smoking cessation and treatment.

## Disease name and synonyms

Respiratory bronchiolitis-associated interstitial lung disease (RB-ILD, ORPHA79127).

The disease has not been referred to by any other synonym in medical literature.

## Definition

Respiratory bronchiolitis-associated interstitial lung disease (RB-ILD) is a rare, mild inflammatory pulmonary disorder that occurs almost exclusively in current or former heavy smokers, usually between the third and sixth decades, most likely with no gender predilection. It is characterized by shortness of breath and cough, pulmonary function abnormalities of obstructive, restrictive, or mixed pattern, and high resolution computed tomography (HRCT) scans showing centrilobular micronodules, ground-glass opacities, and peribronchiolar thickening, often accompanied by tobacco-related centrilobular emphysema [[1],[2]].

RB-ILD is a combined respiratory bronchiolitis (RB) and interstitial lung disease (ILD). The first component of the disease - RB - was first described in 1974 as an incidental histologic lesion in the lungs of young asymptomatic cigarette smokers who died from nonpulmonary causes [[3]]. These pathological changes included accumulation of pigmented macrophages within the bronchioles and the peribronchiolar alveolar spaces in association with minimal chronic inflammation in the bronchiolar walls and the neighboring interstitium, and were rarely found in non-smokers [[3]]. Since that initial report, RB has been recognized as an extremely common histological lesion thought to occur in virtually all cigarette smokers, and the term refers to the universal inflammatory reaction in respiratory bronchioles, known as a smoker’s bronchiolitis [[4]–[6]].

Subsequently, in 1987, Myers et al. described six patients’ smoking histories that had an extent of alveolar accumulation of pigmented macrophages and mural inflammation on surgical biopsy specimens that was severe enough to produce clinical, physiologic, and imaging features of ILD [[6]]. This clinicopathological entity was termed “respiratory bronchiolitis-associated interstitial lung disease” (RB-ILD). Contrary to RB alone, RB-ILD is an uncommon disorder found in a far smaller proportion of smokers [[4],[6]]. Some authors have regarded RB-ILD as an exaggerated RB with more extensive peribronchiolar interstitial fibrosis [[7]–[9]], while others [[4],[6]] have considered the histologic features of RB and RB-ILD indistinguishable and have separated the two based on the presence of clinical evidence of ILD.

RB-ILD has been incorporated into the broad category of diffuse parenchymal lung diseases that are also known as the interstitial lung diseases (ILDs). Within this large heterogeneous group of diseases, mostly of unknown etiology, RB-ILD belongs to a subgroup termed idiopathic interstitial pneumonias (IIPs) [[1],[10]]. The classification of IIPs was first standardized in 2002 by American Thoracic Society (ATS) and European Respiratory Society (ERS) consensus that combined the histopathologic pattern of IIPs seen on lung biopsy with clinical findings in order to establish a final clinicopathologic diagnosis [[1],[11]]. According to a recent update, the ATS/ERS classification of IIPs has abstracted a group of six major IIPs, including RB-ILD, from rare IIPs and unclassifiable IIPs [[10]].

Most insight into RB-ILD to date has been provided by five case series [[6]–[8],[12],[13]] totaling 78 patients with biopsy-proven RB-ILD, These studies have provided descriptions of clinical and physiological features, pathological and radiographic/HRCT appearances, and outcomes of the disease. Additionally, there have been several other reports focused mainly on the differential diagnosis of RB-ILD based on radiological findings [[9]] and their correlation with clinical and physiological findings [[14]], or on follow-up after smoking cessation [[14],[15]].

In spite of the substantial clinical heterogeneity of these studies, it has been consistently reported that patients with RB-ILD are usually ever cigarette smokers with at least 30 pack-years at the time of diagnosis [[6]–[9],[12],[13]]. Only incidental single cases of RB-ILD in non-smokers have been reported, including patients exposed to second-hand smoke [[8],[16]]. The age at symptom onset ranges from 22 to 70 years, with median values of 36–54 years [[6]–[8],[12],[13]]. When the disease occurred at a younger age, it was usually in heavy smokers who had smoked 2 to 3 packs of cigarettes for at least 10 years. However, the issue of gender predilection remains unclear. Some reports have indicated that men were more often affected than women [[6],[9],[12]], while others have reported contradictory data [[13]] or no gender predilection [[8]]. Therefore, the viewpoint that there is no gender predilection seems most likely to be accurate [[2]].

## Epidemiology

The prevalence and incidence of RB-ILD is unknown and has remained difficult to assess for many years. Available epidemiological data on RB-ILD are sparse, and eventually represent a calculation of relative frequency of RB-ILD among ILDs, or more specifically, among IIP cases.

In several earlier case series, the incidence of RB-ILD was usually estimated together with that of desquamative interstitial pneumonia (DIP), and these two IIPs were found to account for 10%–17% of the study samples [[17]–[20]]. RB-ILD was recorded separately in only two of these case series, and accounted for 2% and 13% of cases [[17],[20]].

Several more recent studies have provided additional data on the epidemiology of ILDs under the current ATS/ERS consensus on the classification of IIPs and have even calculated the relative frequency of RB-ILD among them [[21]–[25]]. A wide epidemiological study conducted in Greece found that the prevalence and incidence of RB-ILD were 0.07 per 100,000 and 0.04 per 100,000 [[23]], respectively.

In contrast, in the Saudi Arabian register of newly diagnosed ILD cases, RB-ILD cases accounted for 5.5% of all types of IIPs [[24]]. A German pathology review of ILD cases revealed 9.5% of RB-ILD cases among all types of IIPs [[25]].

## Clinical description

### History and physical

The onset of RB-ILD is usually insidious with exertional dyspnea, symptomatic wheezing, and persistent cough, which may be non-productive, developing over a course of weeks or months [[6]–[8],[13]]. This clinical picture can be masked by concomitant smoking-related chronic bronchitis. RB-ILD may also be diagnosed in asymptomatic patients with functional impairment and chest radiograph or HRCT abnormalities. In most cases, RB-ILD is not a disabling disease and patients display only mild symptoms. However, some patients may have significant dyspnea and hypoxemia because of more extensive ILD [[1]]. Chest pain and weight loss were less frequent and hemoptysis and fever were reported incidentally [[8]], and might have resulted from underlying disease including lower respiratory tract infection [[26]]. In the literature, there is only one case of an acute presentation of RB-ILD with fevers and sweats as general symptoms, and increasing breathlessness and non-productive cough as respiratory symptoms [[27]].

On physical examination, although not universal, bibasilar end-inspiratory crackles are the most common signs. A case series report by Portnoy et al. [[13]], that included a sample size that was twice that of the next largest sample and represented the single largest experience reported to date, showed wheezing as a common sign in 69% of RB-ILD cases, significantly more frequent than in prior reports [[4],[7],[8]].

Digital clubbing was either not reported at all [[6],[7]] or was an uncommon sign with an incidence not exceeding 25% [[8],[12],[13]]. A single reported case of digital clubbing in RB-ILD probably caused by lung cancer that was detected at follow up [[28]] might warrant that occurrence of clubbing in an RB-ILD patient with a typical average of a 30–40-year smoking history should cause the clinician to suspect an occult tumor [[6]].

### Physiologic changes

Pulmonary function tests in patients with minimal symptoms usually reveal a mild to moderate decrease in DLco. In patients with more severe symptoms, both airway obstruction and restriction or occasionally an isolated increase in residual volume may be found [[6]]. These features result from the variable extent of both RB-ILD and centrilobular emphysema in different cases. Patients with more advanced clinical manifestations of disease may have more significant reductions in carbon monoxide transfer [[29]].

The large case series reported by Portnoy et al. showed that an obstructive pattern was the most common pulmonary function defect (47%), while pure restriction or mixed defects were less common (31% and 9%, respectively), and that 13% of patients had normal spirometry data [[13]]. The authors of this study did not support the common reduction in DLco in patients with RB-ILD. Normal initial DLco values were demonstrated in 36% of patients, suggesting lower sensitivity of that marker of clinically significant disease than had been previously indicated [[6]–[8],[12]].

### Natural history and prognosis

Definitive reports of natural history of RB-ILD are not available due to the relatively small number of patients studied to date [[6]–[8],[12],[13]]. A few earlier case series showed that most patients with RB-ILD had a relatively stable clinical course [[12]]. Subsequently, Portnoy et al’s case series on the long-term outcomes of RB-ILD did not support a benign course of RB-ILD, although prolonged survival was still common [[13]]. Symptomatic and physiologic improvements were relatively rare, and neither abstaining from cigarettes nor pharmacologic intervention regularly led to clinically significant benefit. In turn, regardless of smoking status, corticosteroids, or other therapies, self-reported subjective deterioration in overall status or of specific symptoms was commonly observed, as were objective signs of clinical worsening determined by spirometry and gas exchange measurements. Symptoms and physiology improved in only a minority of patients, although the study did agree that prolonged survival could be expected in most patients with RB-ILD and that mortality secondary to progressive ILD is rare.

## Etiology and pathogenesis

RB-ILD corresponds to changes within pulmonary interstitium that are known to occur almost exclusively in current heavy cigarette smokers. However, the pathologic mechanisms that lead to these changes remain unclear, and RB-ILD is regarded as a manifestation of lung parenchymal response to inhaled cigarette smoke in both animals and humans [[30],[31]]. The key feature of this manifestation is the bronchiolocentric accumulation of yellow-brown pigmented macrophages and cellular or fibrotic inflammation with associated clinical features of ILD [[6],[8],[14]]. The pigment in these so called “smokers’ macrophages” represents cigarette smoke constituents, most notably kaolinite (aluminum silicate) [[32]].

RB-ILD is commonly viewed as an amplified respiratory bronchiolitic response that, compared to the solely bronchiole-centered lesions seen in RB, leads additionally to interstitial and air space inflammation and fibrosis extending to the nearby alveoli.

At the same time, RB-ILD has been distinguished from the more exaggerated panlobular diffuse mild-to-moderate interstitial fibrosis and massive accumulation of macrophages in the alveoli described in DIP [[33]]. Both of these uncommon conditions have significant clinical and histopathologic overlap [[34]] and were suggested to be synonymous [[7],[11],[35]]. Together with a common pattern of pathological abnormalities, RB-ILD and DIP show radiological overlap. The only features that may differentiate these two IIPs are the distribution and the extent of the lesions. This observation may again raise the question of whether RB-ILD and DIP represent different points along the spectrum of the same disease [[1],[9],[36]], although RB, RB-ILD, and DIP representing various degrees of severity of the same process caused by long-time tobacco smoking is not commonly considered.

Because of the evidence of the etiological association with cigarette smoking, the latest update of the ATS/ERS classification of IIPs has grouped RB-ILD and DIP together within the major IIPs as smoking-related lung disease [[10]]. However, the pathogenic mechanism that links tobacco-smoke exposure to these entities has not yet been elucidated [[37]].

Although originally postulated by Niewoehner that RB alone could be the precursor of centriacinar emphysema [[3]], it remains largely unclear whether RB-ILD progresses to emphysema, despite a few studies suggesting such a possibility [[38],[39]].

## Diagnosis

It is increasingly accepted that a diagnosis of RB-ILD is secure when based upon typical HRCT findings (ground-glass opacities and centrilobular nodules) in a current smoker, especially when bronchoalveolar lavage (BAL) findings (the presence of smokers’ macrophages and the absence of lymphocytosis) are also compatible. In case of the assumption of an ILD other than RB-ILD, a surgical biopsy may be necessary [[1],[10]].

## Diagnostic methods and criteria

### Histologic features of surgical biopsy specimens

**Figure 1:**
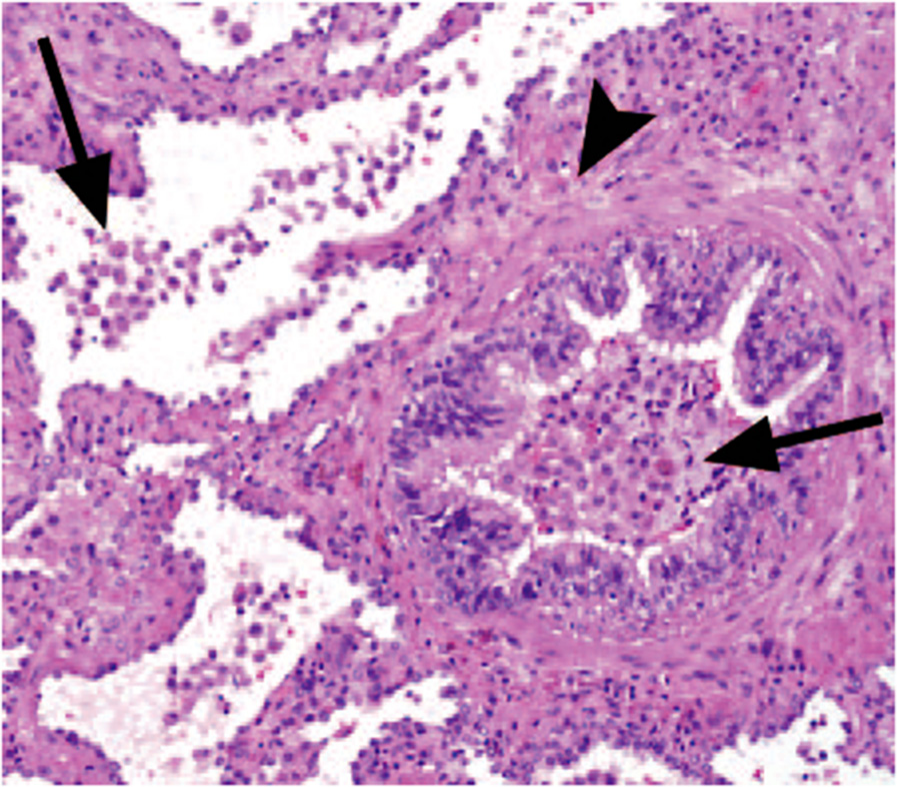
**Photomicrograph (original magnification, x100; hematoxylin-eosin stain) showing the characteristic histologic features of RB-ILD.** Pigmented macrophages in a terminal bronchiole and the adjacent alveoli (arrows), and moderate peribronchiolar inflammation and fibrosis (arrowhead) are present. *Mueller-Mang C, Grosse C, Schmid K, et al.: What every radiologist should know about idiopathic interstitial pneumonias. *Radiographics* 2007, 27:595–615. With permission from The Radiological Society of North America.

According to the international consensus classification of the IIPs, the histopathologic diagnosis of RB-ILD requires the presence of brownish pigmented macrophages within respiratory bronchioles and alveolar ducts, associated with a patchy submucosal and peribronchiolar chronic infiltrate of lymphocytes and histiocytes (Figure 1). The common findings also include mild bronchiolar and peribronchiolar alveolar fibrosis that stretches contiguous alveolar septa and leads to architectural distortion as well as centrilobular emphysema [[1]].

### Findings of bronchoalveolar lavage and transbronchial biopsy

BAL findings in RB-ILD patients usually do not differ from those seen in otherwise normal healthy smokers and include an increased total number of cells with a normal cellular differential analysis [[13]] or an increase in the percentage of macrophages [[15]]. These alveolar macrophages contain brownish, golden, or black tobacco pigment inclusions In the absence of these cells, an alternative diagnosis should be considered. A modest increase in neutrophils may also be present [[40]].

Transbronchial biopsy might be useful in distinguishing RB-ILD from hypersensitivity pneumonitis (HP) or sarcoidosis, but not from DIP. In one case, transbronchial biopsy was sufficient to establish a diagnosis of acute RB-ILD [[27]].

### Radiographic findings

Radiographic findings are usually relatively subtle [[6],[7],[9],[14]]. Normal chest radiographs have been reported in 20% to 28% of patients with histologically-proven RB-ILD [[6],[7],[41]], and in a single case, both normal chest x-ray and HRCT appearance were reported [[41]]. When present, typical chest x-ray findings in patients with RB-ILD may include fine reticulonodular interstitial opacities, which are diffuse [[7]] or are predominant in basal lung areas [[6]]. The chest radiograph may also reveal signs of thickening of the walls of the central and peripheral bronchi [[6],[7],[9]].

**Figure 2:**
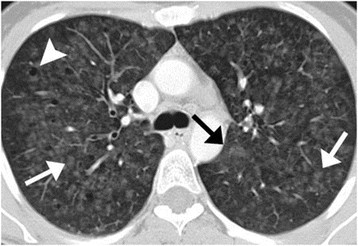
**RB-ILD in a 44-year-old woman with a 20 pack-year smoking history.** HRCT image of the upper lung lobes shows centrilobular nodules (white arrows), patchy ground-glass opacities (black arrow), and mild coexisting centrilobular emphysema (arrowhead). *Mueller-Mang C, Grosse C, Schmid K, et al.: What every radiologist should know about idiopathic interstitial pneumonias. *Radiographics* 2007, 27:595–615. With permission from The Radiological Society of North America.

HRCT findings in RB-ILD patient are shown in Figure 2. Some differences exist between studies in terms of frequency of particular changes and their zonal predominance at HRCT. The most frequent findings reported by Park et al. were central and peripheral bronchial wall thickening (in 90% and 86% of patients, respectively) and centrilobular nodules (71%) [[14]]. Ground-glass opacity was observed significantly less frequently than in the earlier series (67% vs. 100% of patients) [[12]]. Although in a few other studies, the upper lung zones were mostly affected [[8],[9],[14]], Park et al. did not report any zonal predominance. Centrilobular emphysema in the upper lobes was reported consistently as a common (50%–57%) but not severe finding [[8],[9],[14]]. Patchy areas of hypoattenuation were less common (38%) and reported to have lower lung predominance [[14]]. Most probably, this hypoattenuation reflects air trapping due to small airway disease [[14],[42]]. However, a tree-in-bud pattern, which is a common manifestation of small-airway-disease, was rarely reported [[43]]. The frequency of interstitial fibrosis in RB-ILD patients, as determined by the presence of intralobular lines and a reticular pattern, differed significantly in various studies, and ranged from 20% to 75% [[8],[9],[13],[41]], while honeycombing was less common (0%–12%) [[8],[12],[13]].

### Pulmonary function tests

Mixed, predominantly obstructive abnormalities, often combined with hyperinflation and usually associated with a mild to moderate reduction in DLco are the most frequently reported findings in patients diagnosed with RB-ILD [[6]–[8],[13]]. Airflow obstruction is usually mild.

## Differential diagnosis

First, the entities that share a common pathogenesis and clinical-histopathological pattern with RB-ILD, i.e., RB and DIP, should be taken into account in the differential diagnosis. However, separating RB and RB-ILD by sole histopathological criteria remains controversial; moreover, the two entities can also rarely be distinguished solely by the nature and extent of HRCT findings, because there is no arbitrary cut-off point for which the extent of disease at HRCT evolves from RB to RB-ILD [[5]–[9]]. Therefore, it has been proposed that the distinction between RB and RB-ILD should be based not on histological or radiological appearance alone, but on all markers of disease severity taken together, including clinical symptoms, pulmonary function tests results, and HRCT findings. On the contrary, lung biopsy is sometimes required to distinguish RB-ILD from DIP. Although RB-ILD can usually be distinguished from DIP radiologically with HRCT, there is considerable overlap between these two IIPs. In the face of the different prognoses associated with these entities [[7]], correct diagnosis is of considerable importance. For the pathologist, a challenge in the differential diagnosis might be the separation of the fibrotic form of RB-ILD that was suggested by Yousem from DIP [[44]]. This histologic appearance has been linked to a macrophage-poor variant of DIP, and the term RB-ILD with fibrosis has been suggested to represent a subset of diagnoses at the extreme end of the RB-ILD spectrum [[44]]. Further, both RB-ILD and DIP need to be differentiated from airspace enlargement with fibrosis (AEF), the changes which have been incidentally reported on histologic and HRCT examination in smokers, and in comparison to emphysema marked by more extensive interstitial fibrosis [[45],[46]]. In addition, the histopathological distinction between RB-ILD and centrilobular emphysema, which is usually associated with RB and sometimes with interstitial fibrosis, appears to be another challenge. It has been postulated that functional abnormalities might help in making a definite diagnosis [[47]].

Next, other ILDs, including nonspecific interstitial pneumonia (NSIP) and subacute HP, should be taken into account in differential diagnosis. Usually these can be effectively excluded based on the HRCT findings: the ground-glass opacity observed in RB-ILD is usually patchier than that seen in NSIP or acute or subacute HP; additionally, a greater extent of micronodules in acute or subacute HP and the presence of diffuse bronchial wall thickening in RB-ILD may help in differentiating the two entities [[14]]. Additionally, a history of smoking (rare in HP) together with BAL findings (lymphocytosis in HP) can assist in differentiating RB-ILD from subacute HP [[14],[15],[48]].

## Management and disease course

The indications for treatment of RB-ILD are often marginal. Although to date, the regression of findings consistent with RB-ILD after discontinuation of smoking not accompanied by corticosteroid therapy has been reported in few patients [[6],[15],[49]], smoking cessation is considered the most important factor in the management of RB-ILD. Because the majority of patients in reported case series received corticosteroid and/or immunosuppressive therapy along with smoking cessation, it is not yet completely understood whether smoking cessation alone can improve outcomes in RB-ILD [[7],[8],[12],[14],[49]]. It is also unclear whether patients with RB-ILD benefit from corticosteroid therapy with regard to the natural history of the disease. In one report, at follow-up HRCT after corticosteroid treatment and smoking cessation, the extent of bronchial wall thickening, centrilobular nodules, and ground-glass opacities had decreased in 43% of patients, while areas of hypoattenuation had increased as emphysema was irreversible on follow up [[14]]. However, other studies have presented cases with no change or deterioration despite corticosteroid therapy [[8],[12]], or the failure of corticosteroid tapering and discontinuation even in the absence of smoking [[12]].

So far, the prognosis for patients with RB-ILD has been good, with prolonged survival expected in most cases. Until now only one death from progressive lung disease during a median follow-up period of 7 years has been reported during a study that explored the outcomes in 32 RB-ILD patients [[13]]. However, in the absence of other longitudinal data, the median survival time can be only approximate. As was suggested in the study just mentioned, at least 75% of the patients would be expected to live at least 7 years [[13]], although this case series with long follow-up did not support the contention that RB-ILD is a benign disease. Clinical worsening was common, as well as worsening of spirometry results and gas exchange, regardless of smoking status and treatment ordered, including corticosteroids [[13]].

To summarize, the course of RB-ILD is heterogeneous, often with no functional improvement and with disease progression despite smoking cessation and treatment [[8],[12],[13]]. In contrast to previous assumptions, RB-ILD is now less commonly regarded as a benign entity.

## Unresolved questions

1. What are the incidence and prevalence of the RB-ILD?

2. What is the relationship of RB-ILD to other forms of IIPs?

3. What is the relationship of RB-ILD to DIP, and does RB-ILD progress to DIP?

4. What are the potential causes of RB-ILD other than cigarette smoking?

5. Does RB-ILD advance to emphysema?

6. Do smoking cessation and treatment with corticosteroids alter the natural history of RB-ILD?

7. Do any HRCT features of RB-ILD improve following smoking cessation?

## Conclusions

RB-ILD is a rare and not fully understood disease. This clinicopathologic syndrome combines RB and ILD. Because of the evidence of the etiological association with cigarette smoking, RB-ILD is referred to as smoking-related ILD. However, the pathogenic mechanism that links tobacco-smoke exposure to this entity has not yet been clarified, and thus it is still regarded as idiopathic disease. RB-ILD has significant pathological/clinical/radiological overlap with DIP, what raises the question whether RB-ILD and DIP represent different points along the spectrum of the same disease. Further clarification of the relationship between RB-ILD and DIP is warranted. The exact prevalence and incidence of RB-ILD is unknown. Similarly, long-term outcome of RB-ILD awaits recognition. The condition seems not to be as benign as it was previously considered. Some patients respond favorably to corticosteroids and/or smoking cessation, but often the disease progresses despite smoking cessation and treatment. It also remains unclear whether RB-ILD progresses to emphysema.

## Competing interests

The authors declare that they have no competing interests.

## Authors’ contributions

AS and KK participated in the process of the literature review and in the drafting the final manuscript. In addition, AS supervised the project. Authors read and approved the final manuscript.
